# NG2 and phosphacan are present in the astroglial scar after human traumatic spinal cord injury

**DOI:** 10.1186/1471-2377-9-32

**Published:** 2009-07-15

**Authors:** Armin Buss, Katrin Pech, Byron A Kakulas, Didier Martin, Jean Schoenen, Johannes Noth, Gary A Brook

**Affiliations:** 1 Department of Neurology, Aachen University Medical School, RWTH Aachen, Pauwelsstrasse 30, Germany; 2 Centre for Neuromuscular and Neurological Disorders, University of Western Australia, Australia; 3 Department of Neurosurgery, Sart Tilman Hospital, University of Liège, Belgium; 4 Departments of Neurology and Neuroanatomy, University of Liège, Belgium; 5 Department of Neuropathology, Aachen University Medical School, RWTH Aachen, Pauwelsstrasse 30, Germany

## Abstract

**Background:**

A major class of axon growth-repulsive molecules associated with CNS scar tissue is the family of chondroitin sulphate proteoglycans (CSPGs). Experimental spinal cord injury (SCI) has demonstrated rapid re-expression of CSPGs at and around the lesion site. The pharmacological digestion of CSPGs in such lesion models results in substantially enhanced axonal regeneration and a significant functional recovery. The potential therapeutic relevance of interfering with CSPG expression or function following experimental injuries seems clear, however, the spatio-temporal pattern of expression of individual members of the CSPG family following human spinal cord injury is only poorly defined. In the present correlative investigation, the expression pattern of CSPG family members NG2, neurocan, versican and phosphacan was studied in the human spinal cord.

**Methods:**

An immunohistochemical investigation in *post mortem *samples of control and lesioned human spinal cords was performed. All patients with traumatic SCI had been clinically diagnosed as having "complete" injuries and presented lesions of the maceration type.

**Results:**

In sections from control spinal cord, NG2 immunoreactivity was restricted to stellate-shaped cells corresponding to oligodendrocyte precursor cells. The distribution patterns of phosphacan, neurocan and versican in control human spinal cord parenchyma were similar, with a fine reticular pattern being observed in white matter (but also located in gray matter for phosphacan). Neurocan staining was also associated with blood vessel walls. Furthermore, phosphacan, neurocan and versican were present in the myelin sheaths of ventral and dorsal nerve roots axons. After human SCI, NG2 and phosphacan were both detected in the evolving astroglial scar. Neurocan and versican were detected exclusively in the lesion epicentre, being associated with infiltrating Schwann cells in the myelin sheaths of invading peripheral nerve fibres from lesioned dorsal roots.

**Conclusion:**

NG2 and phosphacan were both present in the evolving astroglial scar and, therefore, might play an important role in the blockade of successful CNS regeneration. Neurocan and versican, however, were located at the lesion epicentre, associated with Schwann cell myelin on regenerating peripheral nerve fibres, a distribution that was unlikely to contribute to failed CNS axon regeneration. The present data points to the importance of such correlative investigations for demonstrating the clinical relevance of experimental data.

## Background

The loss of function following human traumatic spinal cord injury (SCI) is often permanent and results in a serious limitation to the patients' quality of life. Despite considerable progress in recent years, the underlying mechanisms responsible for the failure of axonal regeneration after SCI remain only partially understood.

At the spinal cord lesion site, the initial damage to the parenchyma is followed by a complex cascade of secondary events including breakdown of the blood brain barrier (BBB), infiltration of blood-derived inflammatory cells, oedema, excitotoxicity and ischemia. This early phase of secondary parenchymal damage is followed by the removal of tissue debris, resulting in fluid filled cystic cavitation and the deposition of extracellular matrix (ECM) proteins at the lesion epicentre [1]. The surrounding scar is largely composed of astrocytes and a dense, irregular network of their processes. Traumatic injuries which include damage to spinal nerve roots and the meninges also induce fibroblast, meningeal cell and Schwann cell invasion into the lesion site [2-4]. All these cell populations contribute to the production of a dense ECM that presents itself as a molecular barrier to axonal regeneration [1,3,5].

A major class of growth-inhibitory molecules associated with CNS scar tissue is the family of chondroitin sulphate proteoglycans (CSPGs). CSPGs are highly sulphated molecules that consist primarily of one of six possible core proteins, each of which is coated, to varying extents with glycosaminoglycan (GAG) side chains. The CSPGs are expressed throughout the developing CNS, where their axon growth-repulsive properties are believed to play an important role in determining nerve fibre pattern formation. In adulthood, expression levels are generally much lower. The use of broad specificity antibodies in a number of experimental lesion paradigms in adult animals, including cortical injury, nigro-striatal axotomy and SCI, has demonstrated the elevated expression of CSPGs [6,7]. Pharmacological interventions involving the enzymatic degradation of CSPG-GAG side chains have demonstrated a substantially enhanced axonal regeneration and functional recovery following spinal cord injury [8].

Experimental investigations into the role of individual CSPG family members have revealed distinct expression patterns and functions in the traumatically injured CNS. NG2 as an integral membrane proteoglycan shares no homology to other proteins and is present on the surface of oligodendrocyte precursors *in vitro *[9] and in the developing and adult rodent CNS [10,11]. Correlative investigations in human *post mortem *tissue have demonstrated a similar oligodendrocyte precursor distribution in the adult human CNS [12]. NG2 has been reported to undergo a strong up-regulation at the lesion site after experimental traumatic SCI, being detected on oligodendrocyte precursors and invading macrophages [13,14]. Furthermore, treatment with antibodies specifically neutralizing the NG2 proteoglycan has revealed significantly increased axon regeneration following experimental SCI [15].

Neurocan belongs to the lecticans, a sub-division of the CSPG family that is characterized by a hyaluron-binding domain and a C-type lectin domain, both of which enable these molecules to interact with ECM proteins such as tenascin-R [16]. Neurocan is a nervous system-specific CSPG [17] and both *in vitro *and *in vivo *studies point to an axon growth-repulsive role [18,19]. Its presence in regions of active fibre growth during development suggests that neurocan delineates boundaries of neuronal outgrowth and that it may be important for neuronal pattern formation [20,21]. In various CNS lesion paradigms, including SCI, both transient [22], and long term [23] re-expressions of neurocan by astrocytes have been reported.

A second member of the lectican sub-family is versican. This CSPG is also developmentally regulated, being expressed in white matter tracts around the time of myelination [24]. This distribution of versican in the white matter has also been described by others, and an axon growth-inhibitory role suggested [25]. Furthermore, *in vivo *investigations have demonstrated an early up-regulation of this CSPG following experimental lesions, the distribution of which was compatible with a regeneration-blocking effect [22,26]. Cells of the oligodendrocytic lineage were reported to be responsible for the post-traumatic up-regulation of versican following spinal lesions.

Phosphacan is an alternatively spliced variant of the receptor-type protein tyrosine phosphatase. It does not belong to the lectican sub-family of CSPGs but is, nevertheless, able to bind to ECM proteins such as N-CAM and tenascin [27]. In the CNS, its expression peaks during development and in the adult the distribution is more restricted. *In vitro *studies have demonstrated a growth-inhibitory effect of phosphacan on several neuronal populations [28,29]. *In vivo *experiments, following brain and spinal cord injury, have demonstrated an initial decrease in phosphacan concentration at the lesion site [22,23]. However, a marked increase in the expression of phosphacan has been observed in long-term astroglial scars [23].

In contrast to the detailed functional and spatio-temporal information that has been obtained using small laboratory animals, there is relatively little correlative data on the expression of CSGPs following traumatic human spinal cord injury. An immunohistochemical investigation in traumatically injured *post mortem *human spinal cord has demonstrated an up-regulation of CSPGs at the lesion site in human traumatic SCI, but this was spatially correlated with the presence of migrating Schwann cells rather than reactive astrocytes [30]. We have therefore performed a more detailed immunohistochemical investigation on the expression pattern of distinct members of the CSPG family in samples of *post mortem *human spinal cord, taken from patients who died at a range of survival times following severe macerating SCI.

## Methods

*Post mortem*, the spinal cords were removed from 4 control patients who had not suffered from any neurological disease (Table 1) and from 15 patients who died at a range of time points after traumatic spinal cord injury (Table 2). Patients with traumatic injury had been diagnosed as having functionally "complete" injuries and presented clinically with paraplegia or tetraplegia (ASIA A). The study was approved by the Aachen University Ethics Committee. The spinal columns were removed at autopsy, approximately 15–48 hours after death. Following incision of the dura mater, the spinal cord was fixed in 4% paraformaldehyde for at least 2 weeks. Thereafter, blocks of the lesion site and tissue from regions rostral and caudal to the lesion (approximately 1 cm thickness) were embedded in paraffin wax.

**Table 1:** Patients who served as the control group

**Case number**	**Age**	**Cause of death**	**PM fixation time**
1	29 years	Breast cancer	20 hours

2	47 years	Pneumonia	32 hours

3	62 years	Breast cancer	16 hours

4	83 years	Myocardial infarction	45 hours

**Table 2:** Patients who died after traumatic injury to the spinal cord

**Case number**	**Age**	**Injury level**	**Inj.-death interval**	**PM fixation time**
1	21 years	T12	2 days	23 hours

2	51 years	C1	4 days	32 hours

3	84 years	C3–4	5 days	15 hours

4	65 years	C5	8 days	28 hours

5	63 years	C6	10 days	41 hours

6	18 years	T6	11 days	19 hours

7	72 years	T11–12	24 days	25 hours

8	85 years	C3	4 months	22 hours

9	76 years	T8–9	10 months	18 hours

10	80 years	C5–6	1 year	16 hours

11	72 years	T12	2 years	21 hours

12	44 years	L1	8 years	17 hours

13	71 years	C3–4	20 years	18 hours

14	47 years	T5	26 years	20 hours

15	57 years	T3–4	30 years	17 hours

### Peroxidase Immunohistochemistry

Transverse sections (5 μm thick) were collected onto poly-L-lysine-coated slides and allowed to dry. Sections were de-waxed in xylene, rehydrated and unless otherwise stated, were microwaved in 10 mM citrate buffer (pH 6) for 3 × 3 minutes. Sections for neurocan, versican and phosphacan immunohistochemistry were not microwaved; instead they were incubated in 1 U/ml chondroitinase ABC for 2 hours at 37°C. Sections for NG2 were neither microwaved nor treated with chondroitinase ABC. Non-specific binding was blocked by incubation in 0.1 M PBS containing 3% normal goat serum and 0.5% Triton X-100 for 30 minutes. Next, sections were incubated in the primary antibody, overnight at room temperature. The primary antibodies used were: monoclonal mouse anti-human NG2 (clone B5, undiluted cell culture supernatant from ATCC cultures and antibody 9.2.27, diluted 1:50; gift from Prof. R. Reisfeld, Scripps Research Institute), monoclonal mouse anti-neurocan (Chemicon, diluted 1:100), polyclonal rabbit anti-versican (Acris antibodies, diluted 1:1.000), monoclonal mouse anti-phosphacan (Chemicon, diluted 1:500), polyclonal rabbit anti-GFAP (DAKO, diluted 1:2.500), polyclonal rabbit anti-myelin basic protein (MBP) (Chemicon, diluted 1:1.000) and polyclonal rabbit anti-neurofilament (NF, Sigma-Aldrich, diluted 1:2.000).

Following extensive rinsing steps in 0.1 M PBS, sections were incubated in biotinylated horse anti-mouse or anti-rabbit antibody (Vector Laboratories, diluted 1:500) for 1 hour at room temperature. As described earlier, incubation with the biotinylated secondary antibody was followed by the Vector ABC system and a subsequent incubation in diaminobenzidine for visualization of the reaction product. For negative controls the primary antibody was omitted.

### Immunofluorescence

For double immunofluorescence, sections were de-waxed in xylene and rehydrated. Treatment with chondroitinase ABC (1 U/ml) for 2 hours at 37°C (for neurocan, versican and phosphacan immunohistochemistry) was followed by blockade of non-specific binding by incubation in 3% normal goat serum and 0.5% triton X-100 in 0.1 M PBS for 30 minutes and subsequent incubation overnight at room temperature in a combination of the following primary antibodies: monoclonal mouse anti-NG2 (clone B5, cell culture supernatant undiluted and 9.2.27, diluted 1:10), monoclonal mouse anti-neurocan (Chemicon, diluted 1:20), polyclonal rabbit anti-versican (diluted 1:20), monoclonal mouse anti-phosphacan (diluted 1:50), monoclonal mouse anti-neurofilament (NF, Sigma, Clone52, diluted 1:100), polyclonal rabbit anti-NF (diluted 1:1,000), polyclonal rabbit anti-GFAP (diluted 1:1.000), polyclonal rabbit anti-MBP (Chemicon, diluted 1:500) and polyclonal rabbit anti-laminin (Sigma, diluted 1:50). After the subsequent incubation with Alexa 594 (red-fluorescence)-conjugated goat anti-mouse and Alexa 488 (green fluorescence)-conjugated goat anti-rabbit secondary antibodies (Molecular Probes, diluted 1:500) for 3 hours at room temperature, slides were cover-slipped in Moviol. For negative controls, the primary antibodies were omitted.

**Figure 1:**
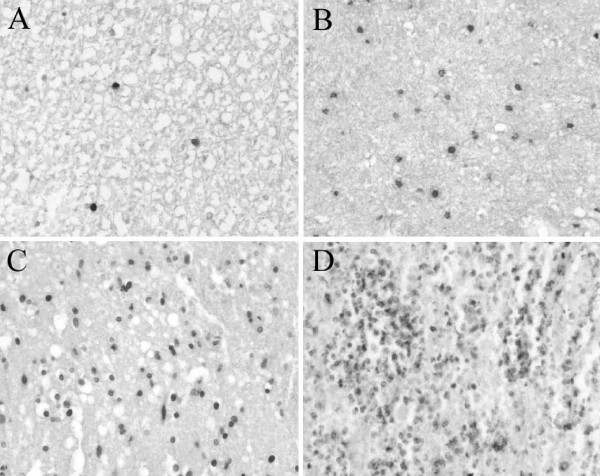
**Examples of CD68-positive macrophage stains obtained from human SCI samples. **The densities of the stained profiles are used to reflect the arbitrary rating scale used for semi-quantification used in this study. Transverse sections of human spinal cords stained for CD68 representing, **A: **a low incidence (arbitrary scale = +) of stained profiles to, **D: **very high incidence (arbitrary scale = ++++) of stained profiles.

For a semi-quantitative description of the amount of NG2-immunoreactive cells detected at the various survival times, an arbitrary rating scale for the number of labelled cells was chosen (see Fig. 1), ranging from 0 (no immunopositive cells) to ++++ (very high incidence of labelled cells).

## Results

The spinal cords of 19 individuals were examined using NG2, neurocan, versican, phosphacan, GFAP, MBP, NF and laminin immunohistochemistry. Both NG2 antibodies revealed an identical staining pattern in control and pathological cases. The brains of all individuals were carefully examined and were declared to be without pathological findings. The spinal cords of the control cases were also without pathological findings. The pathological cases have been sub-divided into two groups according to the post-insult survival times (i.e. early and late survival times), because distinct morphological stages in the formation of the scar were found. For an overview of the results, in particular of the semi-quantitative representation of the number of NG2-immunoreactive cells at the various survival times, see table 3 and Fig. 1.

**Table 3:** Amount of immunopositive cells at different survival times after human SCI

	**Control**	**2–8 days**		**10–11 days**		**24 days**		**4 months**	
		Epicentre	Int. Zone	Epicentre	Int. Zone	Epicentre	Int. Zone	Epicentre	Int. Zone

OPC (NG2)	+++	0	+/++	0	+++	0	+++	0	0/+

CD68	0/+	+/+++	+/++	+++	++	++++	+	0/+	0/+

CD68/NG2	0	+/++	+	++	+	+++	+	0	0

The figures reflect the number of immunopositive cells using an arbitrary rating scale from 0 (no cells) to ++++ (very high incidence of cells) in sections from control spinal cords and the lesion site at various survival times after SCI stained for the different antigens.

### Distribution of NG2, neurocan, versican and phosphacan in the normal adult human spinal cord

**Figure 2:**
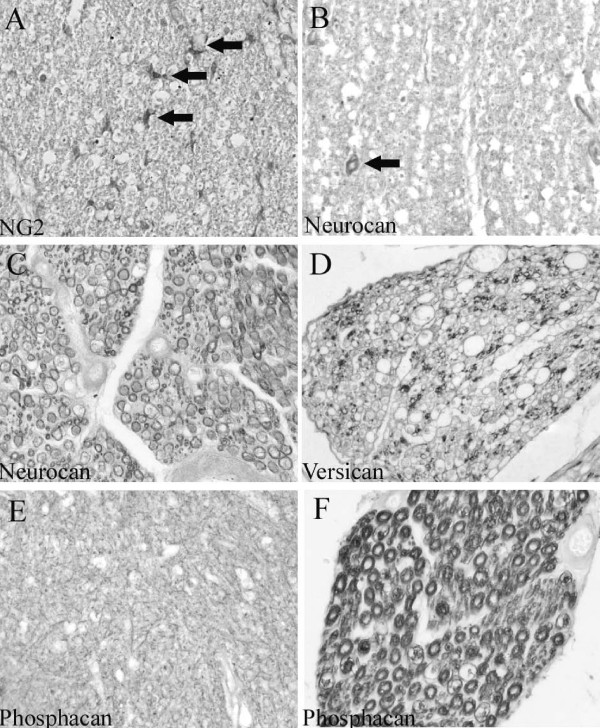
**Normal distribution of NG2, neurocan, versican and phosphacan in the human spinal cord**. Transverse sections of control human spinal cords. **A: **NG2 immunohistochemistry reveals small stellate-shaped cells distributed homogeneously in white matter regions of human spinal cord (arrows). **B: **In the white matter, neurocan immunoreactivity is observed in the wall of a small blood vessel (arrow). Furthermore, a reticular staining pattern can be seen. **C: **In a dorsal nerve root, neurocan staining is present in myelin sheaths. **D: **Versican immunoreactivity is scattered in a dorsal nerve root and can be found in myelin sheaths of small diameter axons. **E: **Phosphacan immunohistochemistry reveals a fine reticular staining pattern in the gray matter. **F: **In a dorsal nerve root, phosphacan-immunopositive myelin rings can be observed. (**A-F **magnification × 320).

**Figure 3:**
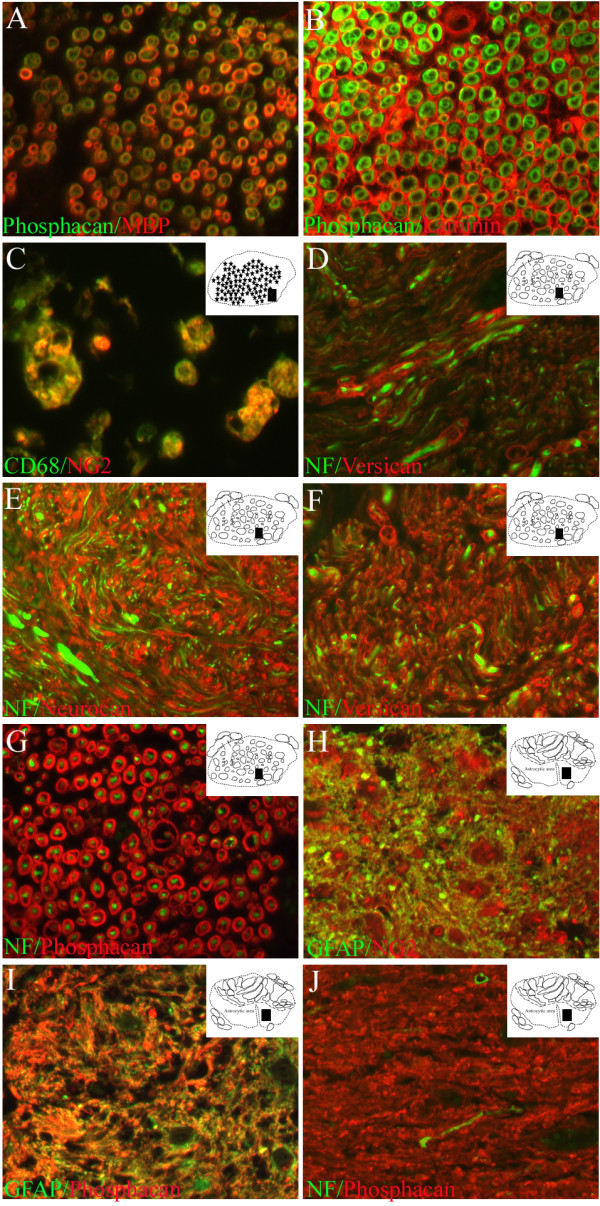
**The cellular and molecular composition of the scar in human SCI after early and long survival times**. Transverse sections of the human spinal cord of control cases and at early and late survival times after SCI. The schematic diagrams in the upper right corner indicate the region from where the actual picture was taken (black rectangle). **A: **Co-localisation of phosphacan (green) and MBP (red) confirms the presence of the CSPG in the myelin sheaths of control dorsal root axons. **B: **Double immunofluorescence with phosphacan (green) and laminin (red) reveals CSPG-immunopositive myelin rings surrounded by laminin-positive endoneurium in a dorsal nerve root of control spinal cord. **C: **Double immunofluorescence with NG2 (red) and CD68 (green) in macrophages at the lesion epicentre, 10 days after injury. **D: **One year after SCI, staining for versican (red) and NF (green) in sections from the lesion epicentre demonstrated individual and bundled regenerated nerve fibres that were surrounded by a versican-positive endoneurium in the ECM. **E-G: **In sections from the lesion epicentre of the same case, double immunofluorescence for NF (green) and neurocan (**E**, red), versican (**F**, red) or phosphacan (**G**, red) revealed nerve fibres surrounded by a CSPG-positive endoneurium. **H-I: **In the intermediate zone of the same case, GFAP (green) and NG2 (**H**, red) and phosphacan (**I**, red) immunofluorescence demonstrated the close overlap of all three proteins in the astroglial scar after human SCI. **J: **In an adjacent section, NF (green) and phosphacan immunohistochemistry revealed occasional, small, nerve fibres still present within the CSPG-rich ECM of the astroglial scar. (**A-J **magnification × 400).

In cervical, thoracic and lumbar segments of the normal, unlesioned spinal cord, NG2 immunoreactivity was restricted to small stellate-shaped cells (Fig. 2A). These cells were evenly distributed in both white and gray matter regions. Neurocan immunoreactivity in the spinal cord parenchyma was observed as a homogeneous, fine reticular pattern in the white matter. Furthermore, many small diameter blood vessels were stained for neurocan (Fig. 2B). In ventral and dorsal nerve roots the myelin sheaths were immunopositive (Fig. 2C). Similar to neurocan, versican immunoreactivity could also be found in a fine reticular pattern in the spinal cord white matter (not shown). In nerve roots, a scattered distribution pattern was detectable in myelin rings surrounding sub-populations of mostly small diameter axons (Fig. 2D). Immunohistochemistry for phosphacan showed a diffuse reticular pattern in both gray and white matter of control spinal cord (Fig. 2E). The staining pattern in ventral and dorsal nerve roots was identical to neurocan with immunoreactivity in myelin sheaths (Fig. 2F). The presence of CSPGs in peripheral myelin was supported by co-localisation with MBP (Fig. 3A). Furthermore, double immunofluorescence with laminin revealed a clear distinction between the CSPG-positive myelin rings and the laminin-positive endoneurium (Fig. 3B).

### Morphological appearance of the lesion site

**Figure 4:**
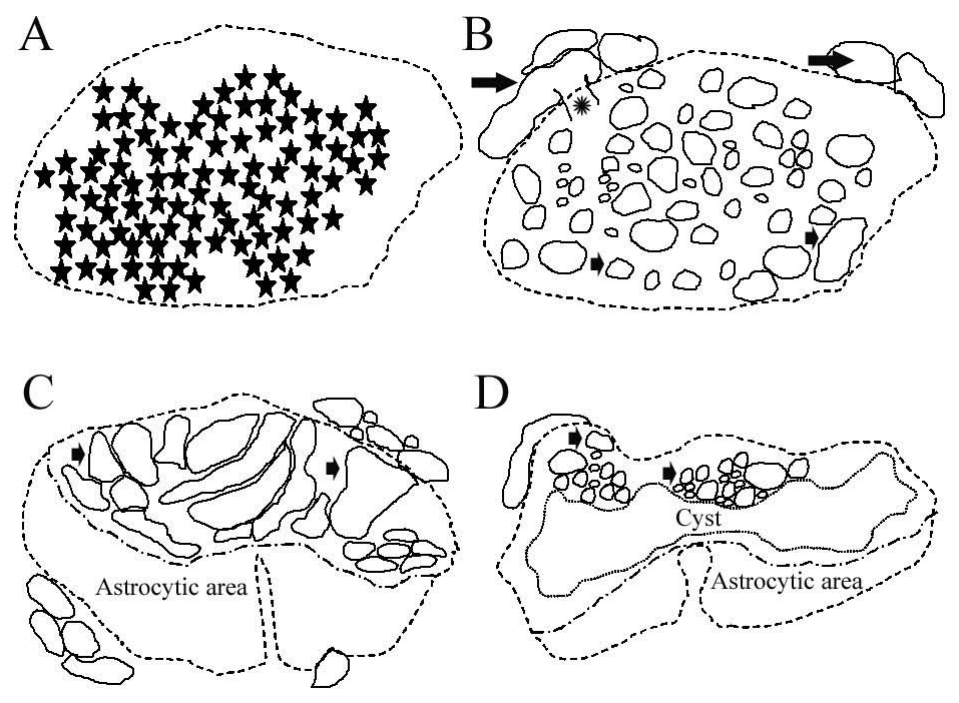
**The typical morphological appearance of the lesion site in severe human macerating SCI**. Schematic diagrams showing the typical transverse appearance of the lesion site in the present cases of severe human traumatic SCI. **A: **At survival times ranging from 2 to 24 days after SCI, the lesion epicentre was characterised by the complete destruction of the cytoarchitecture and a massive hemorrhagic infiltration into the parenchyma (extent of hemorrhage indicated by stars). **B: **At survival times of 4 months and longer after SCI, the lesion epicentre was characterised by numerous regenerated root-like structures (small arrows) of variable sizes embedded in a dense ECM. Furthermore, individual spinal nerve roots (large arrows) and the entry zone of a nerve root into the spinal cord (asterisk) could be seen. **C: **When no cysts were present in the intermediate zone, the lesion was largely divided into an astrocytic scar and the region with nerve root-like structures, including Schwann cells (small arrows). **D**: In the intermediate zone, the lesion could often be sub-divided into a centrally located cystic region surrounded by an astrocytic scar (in this case in the ventral region) and an area with numerous small-medium root-like structures embedded in the ECM of the connective tissue scar (small arrows, in this case in the dorsal region). These schematic diagrams were prepared from representative sections and have been presented to provide a broad indication of where, within sections, particular images have been taken.

The lesion sites of these severe human traumatic SCI cases could be sub-divided into the lesion epicentre and an intermediate zone. For a more detailed description, see Buss *et al*., 2007. Briefly, the lesion epicentre was initially characterised by the complete destruction of cytoarchitecture and massive haemorrhagic infiltration in between amorphous regions of tissue debris (Fig. 4A). At 24 days after injury, Schwann cell migration into the lesion core was seen, and in cases with longer survival times the lesion epicentre was characterised by a dense ECM with embedded nerve root-like structures and individual myelinated nerve fibres (Fig. 4B). However, no astrocytes were detectable in this region.

The intermediate zone included the extremities of the lesion site and their interface with the adjacent damaged, but non-degenerating, CNS parenchyma. At 24 days after injury, the remaining astrocytes appeared activated and produced a dense, irregular scar. At longer survival times, the intermediate zone showed a clear demarcation between the Schwann cell area and the astroglial scar (Fig. 4C). In most cases, cystic cavitation could also be seen in between the 2 areas (Fig. 4D).

### Early survival times (2 to 11 days post insult)

#### Lesion epicentre

**Figure 5:**
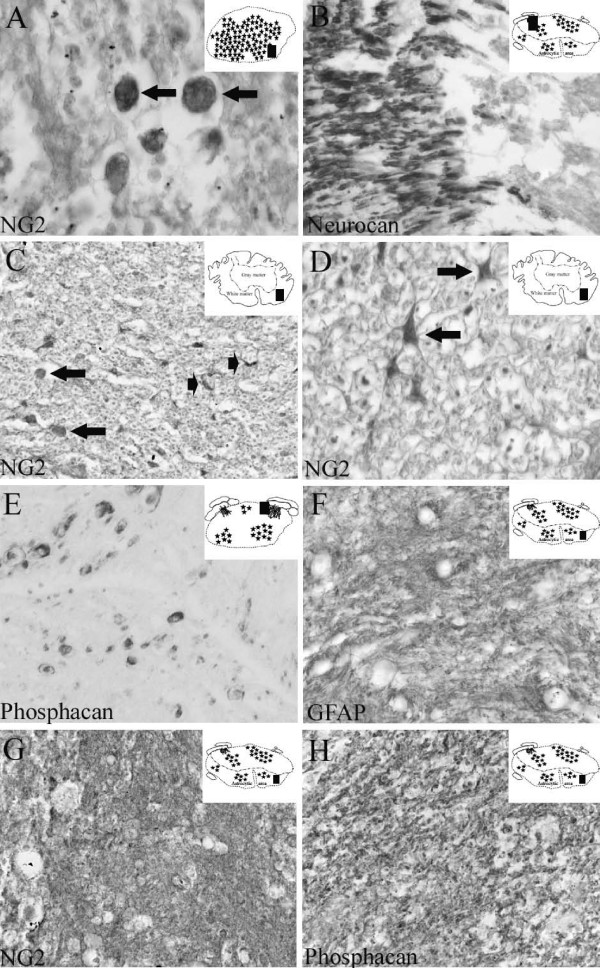
**The cellular and molecular composition of the scar following human SCI at both early and long survival times**. Transverse sections of the human spinal cord of patients with survival times of 10 to 24 days after SCI. The schematic diagrams in the upper right corner indicate the region of the section from which images were prepared (black rectangle). **A: **Ten days after SCI, NG2 staining demonstrated round to oval-shaped cells at the lesion epicentre (arrows). **B: **In sections from the lesion site of the same case, neurocan immunoreactivity revealed myelin staining in a dorsal nerve root that stopped abruptly at the dorsal root entry zone. **C: **Eleven days after SCI, NG2 staining in the intermediate zone demonstrated cells with a round to oval morphology (arrows) and small stellate-shaped cells (arrowheads). **D: **In the same section, higher magnification better illustrated the stellate-shaped morphology of oligodendrocyte precursor cells (arrows). **E: **Twenty-four days after SCI, phosphacan staining was present on myelin rings of some axonal structures close to the lesion epicentre. **F: **In sections from the intermediate zone of the same case, GFAP immunohistochemistry revealed a dense network of irregular fibres without identifiable cell bodies in between. **G-H: **In near adjacent sections from the intermediate zone, NG2 (**G**) and phosphacan (**H**) staining revealed similar distributions, with a dense, irregular network of fibres and no clearly identifiable immunoreactive cell bodies. (**A-C **and **E-H **magnification × 320, **D **magnification × 640).

In the present *post mortem *cases, the lesion epicentre was characterised by the complete destruction of cytoarchitecture. From 2–8 days after trauma, no specific staining for NG2, neurocan, versican or phosphacan could be detected in this area (not shown). At 10 and 11 days after SCI, immunohistochemistry for NG2 revealed cells with a round to oval morphology at the lesion core (Fig. 5A). Subsequent double immunofluorescence demonstrated that these cells were macrophages (Fig. 3C). It was not possible to determine if these cells actually expressed NG2 or were immunoreactive due to phagocytosis of NG2-containing debris from the lesioned parenchyma.

#### Intermediate zone

In the less severely affected areas at the border of the lesion epicentre, the density of astrocytic cells and their network of processes was dramatically decreased compared to control cases (not shown). In accordance with the observations at the lesion core, immunohistochemistry for neurocan, versican and phosphacan revealed no immunoreactive structures in the CNS parenchyma. At the PNS-CNS interface, the immunopositive PNS myelin staining for all 3 CSPGs, stopped abruptly (Fig 5B). NG2 staining was found in some round to oval-shaped cells in the intermediate zone. Furthermore, some stellate-shaped cells were NG2 immunopositive, however their numbers were initially reduced by 2–8 days post injury in comparison to control cases but had returned to near normal levels by 10–11 days after SCI (Table 3 and Fig. 5C–D).

### Moderate to late survival times (24 days to 30 years post insult)

#### Lesion epicentre

At 24 days after trauma, Schwann cell migration was detected within the damaged spinal parenchyma in the vicinity of spinal nerve roots. These cells infiltrated up to 900 μm into the spinal cord tissue with a decreasing density towards the more central regions. Furthermore, outside the CNS parenchyma, root-like structures of various sizes could be found around the original nerve roots [31]. These structures, with an often more complex and convoluted appearance in comparison to nerve roots in control tissue demonstrated scattered immunoreactivity for neurocan, versican and phosphacan. At the 24 days survival time, only occasional axons in the neuromas were surrounded by neurocan, versican or phosphacan immunopositive myelin rings (Fig. 5E). NG2 was similar to that seen at 10 and 11 day survival times, with round to oval cells scattered around the lesion core (not shown). No immunoreactivity could be found in the root-like structures.

**Figure 6:**
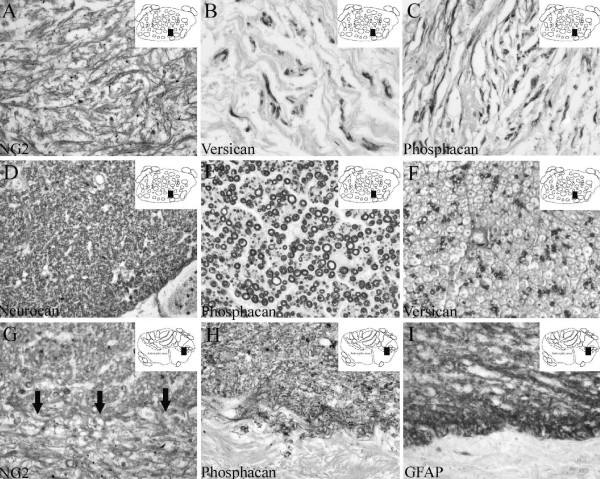
**The cellular and molecular composition of the scar in human SCI after long survival times**. Transverse sections of the lesioned human spinal cord at 1 year (A-C) and 20 years (D-I) after injury. The schematic diagrams in the upper right corner indicate the region from where the actual picture was taken (black rectangle). **A: **NG2 staining revealed a network of irregular lamellae in the ECM at the lesion epicentre. **B-C: **In near adjacent sections, versican (**B**) and phosphacan (**C**) immunohistochemistry demonstrated fibre-like structures either singly or in small bundles. **D: **Twenty years after SCI, nerve root-like structures at the lesion epicentre demonstrated neurocan-positive myelin rings. **E: **In a near adjacent section, phosphacan immunoreactivity was also present in myelin sheaths surrounding axons in neuromas. **F: **Versican immunopositive myelin rings surrounding regenerated nerve fibres were more scattered and of a small diameter in the nerve root-like structures at the lesion epicentre. **G: **Twenty years after SCI, diffuse but elevated levels of NG2 immunoreactivity were still associated with the ECM of the connective tissue scar (lower part of image) as well as with the astroglial scar of the intermediate zone (upper part of image). At the lesion epicentre, NG2 staining was located on more loosely arranged sheets of basal lamina-like ECM. In the astrocytic scar, NG2 was associated with a dense irregular network of processes. Arrows demarcate the border between the connective tissue component of the lesion and the adjacent astrocytic scar. **H: **In a near adjacent section, diffuse phosphacan immunoreactivity was also associated with the dense network of processes of the astroglial scar. No immunoreactivity was associated with the ECM of the connective tissue scar. **I: **GFAP staining strictly delineated the interface between the astroglial scar and the connective tissue scar. (**A-I **magnification × 320).

In sections from cases with survival times of 4 months and longer, the lesion site could be clearly divided into two regions: (i) a Schwann cell-containing area (which could be further sub-divided into areas rich in ECM or in neuromas) and (ii) an astrocyte-dominated scar. The lesion epicentre, at this survival time, revealed massive infiltration by Schwann cells (not shown). This area had, by now, become partially filled with sheet-like lamellae of extracellular matrix which were immunopositive for NG2 but did not stain for neurocan, versican and phosphacan (Fig. 6A). In between this matrix however, irregular fibre-like structures were stained for neurocan, versican and phosphacan (Fig. 6B–C). Double immunofluorescence revealed these to be myelin sheaths around nerve fibres which had regenerated throughout the ECM of the lesion epicentre, either singly or in small bundles (Fig. 3D). At the longer survival times, the round and oval root-like structures resembling re-growing processes of nerve root fibres could also be seen in the former spinal cord parenchyma, now infiltrated by non-CNS cells such as Schwann cells. These neuromas contained larger numbers of Schwann cells and myelinated nerve fibres. The myelin sheaths also contained CSPGs. Immunohistochemistry for neurocan and phosphacan revealed a dense staining pattern in myelin rings of both proteins in the root-like structures (Fig. 6D–E and Fig. 3E, G). Immunohistochemistry for versican also revealed immunopositive myelin sheaths. Its distribution, however, was more heterogeneous, being associated with small diameter axons (Fig. 6F and Fig. 3F).

#### Intermediate zone

At 24 days after SCI, the intermediate zone was devoid of infiltrating Schwann cells. Instead, the first signs of astrocytic scar formation were visible [31]. In these regions of gray and white matter, a densely packed mass or network of diffusely stained GFAP-positive processes could be seen (Fig. 5F). NG2 and phosphacan immunohistochemistry revealed a staining pattern very similar to GFAP with a dense, irregular pattern of processes without identifiable cell bodies (Fig. 5G–H). At survival times of 4 months and longer after SCI, the territory of the densely packed GFAP-positive astroglial scar was clearly distinguishable and distinct from that of the Schwann cell dominated lesion core. At these survival times, NG2 staining revealed a dense, mostly diffuse staining pattern in the astrocytic scar. No clearly identifiable immunopositive cell bodies could be found in this area. Phosphacan immunohistochemistry also revealed a similar distribution to that of GFAP-positive astrocytes, also without clearly identifiable cell bodies (Fig. 6G–I). Double immunofluorescence confirmed the nearly identical distribution patterns of both NG2 and phosphacan with that of the reactive GFAP-positive astroglia in this area (Fig. 3H–I). In the astroglial scar, occasional, single thin nerve fibres could be found within the CSPG-rich ECM (Fig. 3J). At no survival time could neurocan- or versican-immunopositive structures be found in the intermediate zone of human SCI (not shown).

## Discussion

Investigations using experimental animals have demonstrated a central role for CSPGs in the lack of regeneration after SCI. Most members of this proteoglycan family have demonstrated up-regulation after spinal injury, being prominent in the astroglial component of the scar [13,14,22]. Furthermore, the application of chondroitinase ABC (an enzyme capable of degrading GAG side chains) after a spinal cord contusion injury has resulted in increased nerve fibre outgrowth associated with enhanced functional recovery [8]. The application of NG2-specific antibodies following transection injuries to the rat spinal cord has led to a significant improvement in the regeneration of lesioned dorsal column axons [15]. Despite the clear identification of the re-expression of numerous CSPGs in experimental animal models of traumatic injury and the demonstration, both *in vitro *and *in vivo*, of the axon-growth repulsive effect of these molecules, there have been relative few correlative investigations demonstrating the expression of such functionally important molecules in *post mortem *human nervous tissue following injury. In one such publication, a broad specificity CSPG antibody (CS-56, Sigma) was used to demonstrate that the lesion induced distribution of this proteoglycan family was not associated with the evolving astroglial scar. It was, however, associated with migrating Schwann cells and regenerating peripheral nerve fibres within the lesion epicentre. Such a distribution did not point to a central role for CSPGs in the growth-inhibitory milieu of the glial scar after human SCI [30].

In the present investigation, the expression pattern of 4 individual members of the CSPG family was studied in patients who died at various survival times after SCI. In contrast to the previous studies, which used a general immunohistochemical marker for CSPG, the present investigation demonstrated that NG2 and phosphacan were indeed present in the lesion-induced astroglial scar, supporting the notion that these molecules may contribute to the lack of axonal regeneration following human SCI. This data strongly suggests that monoclonal CS-56 antibody may not be capable of detecting, with sufficient sensitivity, all members of this protein family in wax embedded sections.

In sections from control spinal cord, NG2 immunoreactivity was restricted to stellate-shaped cells corresponding to oligodendrocyte precursor cells. This pattern was identical to a previous study in unlesioned human CNS using several antibodies including the 9.2.27 antibody [12]. The distribution patterns of phosphacan, neurocan and versican in control human spinal cord parenchyma were similar, with a fine reticular pattern being observed in white matter (but also located in gray matter for phosphacan). Neurocan staining was also associated with blood vessel walls. This distribution pattern is identical to a previous study in human brain tissue [32]. Although experimental studies have demonstrated an interaction of CSPGs with proteins involved in nerve fibre organisation [33] and phosphacan has been shown to interact with neuronal cell adhesion molecules [34], the exact function of these proteins in the normal CNS remains unknown. In contrast to the present investigation, showing the presence of phosphacan, neurocan and versican in the myelin sheaths of control and regenerating ventral and dorsal nerve roots axons, others have reported a distribution pattern associated with nerve root endoneurium [32]. However, the co-localisation of certain CSPGs with myelin is not without precedent. Others have clearly demonstrated the association of brevican and V2 versican with a myelin fraction extracted from porcine spinal cord. Furthermore, much of the axon growth inhibitory capacity was attributed to these CSPGs [4,25].

After SCI, the distribution pattern of all four CSPGs changed significantly. At survival times of 2 to 8 days after injury, no NG2 immunopositive structures were detectable at the lesion epicentre. However, by 10–24 days, NG2-positive cells could, once again, be identified at the lesion core as well as in the intermediate zone. At the lesion epicentre, double immunofluorescence of such NG2-positive cells revealed that they were macrophages. In the intermediate zone, both round to oval shaped macrophages and stellate-shaped oligodendrocyte precursor cells were NG2 immunopositive. Although others have reported NG2-expression by Schwann cells, it is unlikely that such cells contributed to the NG2 signal observed in the present investigation, since an earlier report from our group using the same human SCI cases was unable to find any evidence of Schwann cell migration in this particular region with an anti-NGFr antibody [31]. An experimental spinal cord contusion injury model has demonstrated NG2-positive non-myelinating Schwann cells, oligodendrocyte precursor cells and macrophages at the lesion site [35]. NG2 was reported to be largely present in the ECM at the lesion core, which contained numerous migrating fibroblasts and Schwann cells. The NG2-positive lamellae were closely associated with P0-myelinated axons. In accordance with these results, the present human SCI cases also demonstrated NG2-positive lamellae in the lesion epicentre. Regenerating nerve fibres, originating from injured spinal nerve roots [31] were embedded within the NG2-positive ECM. It is possible that these lamellae might have played a supporting role in the growth of PNS axons from lesioned nerve roots.

The post lesion survival times of the cases used in the present investigation indicate that the deposition of an NG2-positive matrix took place between 24 days and 4 months after human SCI. The spatial distribution correlates with the animal data [15], however the timing of NG2-expression in traumatically injured human spinal cord is substantially delayed [13,35]. In the present investigation, only macrophages located at the lesion epicentre were found to be immunoreactive and, thus, may have been responsible for NG2 deposition. As described by others, migrating Schwann cells or oligodendrocyte precursor cells (OPCs) may also have contributed to the CSPG-rich ECM [34]. However, the present investigation was not able to give any direct evidence to support this notion. It is possible that sub-optimal antigen preservation or antigen retrieval may have been responsible for an incomplete representation of NG2 distribution. The inevitable delays that occur before human *post mortem *tissues can undergo fixation certainly contribute to this problem. NG2 was, nonetheless, clearly detectable in macrophages and in the ECM of both the Schwann cell-dominated area as well as in the astroglial scar being devoid of Schwann cells.

In the present material, no phosphacan-positive structures could be seen at the lesion epicentre or in the intermediate zone at early survival times of up to 11 days after SCI. Twenty-four days after injury, the evolving astroglial scar in the intermediate zone contained phosphacan with a dense network of irregularly distributed fibres. This distribution, which corresponded to that of astroglial GFAP was seen in all cases with survival times longer than 24 days after SCI. This temporal and spatial expression pattern suggests that reactive astrocytes may have been responsible for the increased phosphacan expression following human SCI. Such a distribution following experimental spinal cord injury has already been suggested by others [22,23]. However, since no clearly identifiable phosphacan-positive cell population could be found, the astroglial origin of this proteoglycan following human SCI must remain speculative. Together with NG2, phosphacan may represent an important growth-inhibitory molecule that is associated with the developing astroglial scar and thus, may be of functional importance for the inhibitory glial environment following human traumatic SCI.

Apart from being associated with the astrocytic scar, phosphacan was also present in the lesion epicentre with a distribution pattern that corresponded to neurocan and versican. This lesion area contained migrating fibroblasts, meningeal cells and migrating Schwann cells and was strictly separated from the surrounding astrocytic scar [31]. All three CSPGs were associated with the myelin sheaths of nerve fibres that were mostly arranged in root-like structures and were derived from regenerating spinal nerve roots [31]. Furthermore, no nerve fibres were detected traversing the interface between the lesion epicentre and the astroglial scar. Therefore, the distribution pattern of neurocan and versican at the lesion epicentre in the present *post mortem *human material is not able to support the notion that either molecule contributes to the failure of CNS axon regeneration after human SCI. However, in the absence of definitive proof of the peripheral source of all nerve fibres within the lesion core, it cannot be excluded that some Schwann cells and their myelin sheaths including the CSPGs were associated with re-growing CNS axons. The function of CSPGs in the myelin sheaths surrounding PNS nerve fibres at the lesion core is currently not known.

## Conclusion

The involvement of four members of the CSPG family has been demonstrated in the post-traumatic events after human SCI. This investigation comprised of 15 patients who died at a range of different survival times after trauma. Due to the difficulties in obtaining *post mortem *human tissue specimens, only one case could be studied at each survival time. Although cases with similar survival times demonstrated similar immunohistochemical staining patterns, the present results need to be interpreted with caution, and further studies with more human cases per survival time would be of significant value.

The present data extends our investigations into the cellular and molecular composition of the lesion site of severe macerating human SCI [31,36,37]. The distribution pattern of individual members of the CSPG family varies significantly after human SCI. NG2 and phosphacan were both present in the evolving astroglial scar and, therefore, might have played an important role in the blockade of successful CNS regeneration. Neurocan and versican, however, were located at the lesion epicentre, associated with Schwann cell myelin on regenerating peripheral nerve fibres, a distribution that was unlikely to contribute to failed CNS axon regeneration. This interpretation certainly needs to take into account the limited number of time points studied after human SCI. It is possible that transient up-regulation of various CSPGs may have been missed due to a lack of appropriate specimens at these time points. The present data emphasises the importance and potential usefulness of comparing data obtained with experimental animals with that obtained from human *post mortem *tissues. This is of particular relevance for the identification of possible key functional molecules that are believed to play a major role in the failure of axonal regeneration following traumatic human spinal cord injury.

## Abbreviations

BBB: Blood brain barrier; CNS: Central nervous system; CSPG: Chondroitin sulphate proteoglycan; ECM: Extracellular matrix; GFAP: Glial fibrillary acidic protein; GAG: Glycosaminglycan; MBP: Myelin Basic Protein; NF: Neurofilament; OPC: Oligodendrocyte Precursor Cell; PNS: Peripheral nervous system; SCI: Spinal cord injury.

## Competing interests

The authors declare that they have no competing interests.

## Authors' contributions

AB designed and coordinated the study and drafted the manuscript. KP carried out most of the immunohistochemical stainings. BK participated in the design of the study, provided specimens and helped to draft the manuscript. DM and JS participated in the design of the study, provided specimens and helped to draft the manuscript. JN participated in the design of the study and helped to draft the manuscript. GB participated in the design and coordination of the study and helped to draft the manuscript. All authors read and approved the final manuscript.

## Pre-publication history

The pre-publication history for this paper can be accessed here:


